# Effect of metronidazole on placental and fetal development in albino rats

**DOI:** 10.21451/1984-3143-AR2018-0149

**Published:** 2019-11-18

**Authors:** Welma Emídio da Silva, Ismaela Maria Ferreira de Melo, Yuri Mateus Lima de Albuquerque, Aline Ferreira da Silva Mariano, Valéria Wanderley-Teixeira, Álvaro Aguiar Coelho Teixeira

**Affiliations:** 1 Universidade Federal Rural de Pernambuco, Departamento de Morfologia e Fisiologia Animal, Recife, PE, Brasil

**Keywords:** metronidazole, pregnancy, placental development, reproduction

## Abstract

Metronidazole is an antiprotozoal and antibacterial used in gynecology and obstetrics for the treatment of parasitic infections. However, despite having clinical use for more than three decades, questions about the safety of its use during pregnancy is not well understood. Thus, the present study evaluated the effect of metronidazole on placental and fetal development in pregnant rats. Metronidazole was orally administered by gavage at a dosage of 130 mg/kg for 7 and 14 days. Morphological analysis, morphometry and immunohistochemistry were performed at the implantation sites and placentas with 14 days of development. The results showed that in the treated group there was a significant reduction in the number of implantation sites, total placental disc area and constituent elements of the labyrinth and spongiotrophoblast layers. Histochemical analysis revealed no significant changes in the content of collagen, elastic and reticular fibers. The TUNEL test showed apoptotic activity in the implantation sites and placentas with 14 days of development independent of the treatment. There was no evidence of malformation in the neonates. However, there was a significant reduction in the number and weight of neonates in the group treated with metronidazole when compared to the control group. Thus, it is concluded that the administration of 130 mg/kg of metronidazole during pregnancy in rats, in addition to interfering with the number of implanted embryos, promotes changes in placental structure and interferes with fetal development. This suggests that this drug should be used with caution during pregnancy.

## Introduction

Metronidazole is a synthetic agent belonging to the class of nitroimidazoles with clinical applicability in both human and veterinary medicine. Since 1959, it became a drug widely used to treat bacterial infections and several protozoa (Vicente and Pérez-Trallero, 2010; Agarwal et al., 2016), also being used in the process of tecidual regeneration (Trindade et al., 2010; Ho et al., 2017).

In gynecology and obstetrics, this medication is prescribed to treat infections such as vaginitis caused by Trichomonas vaginalis, bacterial vaginosis and mixed pelvic infections (Vicente and Pérez-Trallero, 2010), pathologies that have acquired importance in obstetrics due to their direct correlation with cases of preterm labor, premature rupture of membranes, low weight concepts and chorioamnionitis (Souza et al., 2012).

Although the beneficial effects of metronidazole are well documented in the literature (Gülmezoglu and Azhar, 2011; Souza et al., 2012), the safety of the use of metronidazole during pregnancy has not yet been well established. According to Ribeiro et al. (2013), the use of medication in pregnancy and lactation has always been a challenge for health professionals, since it implies potentially damaging action not only for women, but also for the fetus.

In this context, the US Food and Drug Administration adopted the classification of drugs according to the risk associated with their use during pregnancy and classifies them according to the degree of pregnancy risk (Sahin et al., 2016). This classification is predominantly based on the first trimester of pregnancy, since it is during this gestation period that the main embryological transformations occur and being the one with the greatest risk of harmful action (Irvine et al., 2010; Mitchell et al., 2011).

Metronidazole belongs to the pharmacological class of drugs of the B risk category for pregnancy, which indicates that there is no evidence of damage to fetuses, but there are no adequate and well-controlled studies in pregnant women (Irvine et al., 2010). It is also known that this heterocyclic compound is widely distributed and reaches all tissues and body fluids (Vicente and Pérez-Trallero, 2010) and can cross the placental barrier as well as penetrate the fetal circulation and amniotic fluid (Thompson et al., 2016).

In addition, research conducted with the administration of this post-implantation drug indicated embryolethality in rats (Mudry et al., 2001). Kazy et al. (2005) and stated that there is a possible association between vaginal metronidazole, used during pregnancy, and congenital hydrocephalus. Shennan et al. (2006) conducted a randomized study based on the use of antibiotics during pregnancy and concluded that preterm birth may be increased by metronidazole therapy. The use of this drug during pregnancy was also associated with an increase in spontaneous abortions (Muanda et al., 2017).

With these data, the hypothesis of a possible damaging action of metronidazole to the fetus is developed and more research is needed to clarify the possible risks of exposure of the embryo/fetus to this 5-nitroimidazole derivative. Therefore, the aim of the present study was to analyze the effect of administration of metronidazole during pregnancy on placental and fetal development in pregnant rats.

## Methods

This work was performed in the Laboratory of Histology of the Federal Rural University of Pernambuco (UFRPE) after approval (23082.010870/2010) by the Ethics Committee on the Use of Animals - UFRPE. Thirty 90 days old adult female albino rats (*Rattus norvegicus albinus*) with approximately 200g of the Wistar strain were used from the Bioterium of the Department of Animal Morphology and Physiology (UFRPE). The animals were housed in cages and maintained with food and water ad libitum, at ambient temperature of 22°C ± 2 with artificial light produced by fluorescent lamps (Phillips brand, daylight model, 40W), which established the 12-hour light/dark photoperiod, considering the light period was from 6:00 a.m. to 6:00 p.m.

After an adaptation period, vaginal smears were collected to determine the estrous cycle. Females that had at least three regular cycles were mated and randomly divided into two groups, each composed of 15 animals (number acceptable for statistical analysis): Group I - pregnant rats without treatment and Group II - pregnant rats treated with metronidazole. For mating, two females were placed with one male (healthy animals were used for breeding and were coming from the same bioterium) in each box and they remained together for a period of 12 hours (6:00 p.m. to 6:00 a.m.). In the morning of the following day, colpocytological examinations were performed to confirm the mating. The presence of spermatozoa in the vaginal smears was the indicative of the first day of gestation (DG 1) and these females were kept in individual boxes until the day of euthanasia.

Metronidazole was administered orally (gavage) after the mating confirmation at the daily dosage of 130mg/kg in 0.5mL of physiological solution for 7 and 14 days. The dosage was established according to Mudry et al. (2007), differing only in the route of administration. Group I received only the vehicle (physiologic solution) by the same route and period.

Five females from each group were euthanized on the seventh and fourteenth day of pregnancy for analysis of the implantation sites and placentas, respectively. For this, females were anesthetized with ketamine hydrochloride (80mg/kg) and xylazine (6mg/kg) intramuscularly. An exploratory laparotomy with exposure of the uterine horns was performed to count implantation sites, resorption sites and fetuses (Medeiros et al., 2014). The uterine horns were then removed, fixed in 10% formaldehyde and processed for inclusion in paraplast. Then, the anesthesia was deepened to the lethal dose. The paraplast blocks were cut with a Minot type microtome (Leica RM 2035) adjusted to 5mm. The histological sections obtained were placed on slides with Mayer's albumin and kept in a regulated oven at 37°C for 24 hours for drying. The cuts were submitted to hematoxylin-eosin (HE) staining, Mallory's trichrome, nitric orcein and silver impregnation, then analyzed with an OLYMPUS BX-49 light microscope and photographed in an OLYMPUS BX-50 photomicroscope.

Five remaining females from each group were studied throughout the gestation, where the GII group received metronidazole from day 1 (DG 1) to 21 (DG 21). The one day old neonates were counted, weighed in analytical balance and measured with the aid of a pachymeter. Later they were examined for conformation of the eyes, mouth, skull, anterior limbs, hind limbs and tail, implantation of the ears and formation of the anal orifice to investigate possible malformations and/or external abnormalities.

For morphometric analysis of the placentas, ten slides from groups I and II were used and placental disc regions were analyzed. Measurements were restricted to the regions of the labyrinth, trophosphage and giant trophoblastic cells. The images were captured using a Sony® Video Camera attached to the Olympus® Bx50 microscope. Morphometry was performed using the Line Morphometry application, calibrated in micrometers, associated with the Optimas® 6.2 program for Windows.

Morphometry analysis was obtained by the quantification of points with a 110 points graticule, with the following being counted: intervillous maternal blood spaces, fetal vessels and syncytiotrophoblast cells in the labyrinth region; the trophoblastic cells and the trophoblasts in the spongiotrophoblast region, as well as the giant trophoblastic cells, were analyzed in the 40X objective, where 15 fields per placental region were randomly selected.

For the detection of apoptotic cells, the TUNEL technique was used. To do so, the sections were dewaxed in xylene and rehydrated through gradual ethanol washes and the endogenous peroxidase activity was blocked with 0.3% hydrogen peroxide (H2O2) for 30 min at room temperature. Proteinase K was then applied for 15 minutes and sections were washed in PBS (pH 7.4). The cuts were then incubated in TdT (terminal deoxynucleotide transferase) at 37° for 60 minutes in a humid chamber. The stop solution was applied for 10 minutes at room temperature, then the slides were washed in PBS (pH 7.4) and incubated in anti-digoxigenin. Then, the slides were rinsed in PBS (pH 7.4), revealed with the chromogenic substrate diaminobenzidine (DAB, Dako CytomationTM), counterstained with hematoxylin, dehydrated in increasing alcohol concentrations and cleared in xylene. The apoptotic index was determined by counting the percentage of positive cells from at least 500 nuclei subdivided into 10 randomly chosen fields using the 40X objective (Wu et al., 2013).

Differences between the two groups for the implantation site quantification, number of neonates and their respective weights and sizes, as well as measurements of the placental disc and the apoptotic index were analyzed with Wilcoxon-Mann-Whitney nonparametric test. Diffrences were considered significant when p<0.05.

## Results

Statistical analysis of the number of implantation sites means in the experimental groups revealed that treatment with metronidazole significantly reduced sites number relative to control (Table 1).

**Table 1:** Number of sites of implantation (7 days), placenta weight (g) at 14 days of gestation and total area (μm2) of the placental disc of experimental groups's rats. The results represent Means ±SEM of groups of 15 rats^*^.

	**GI**	**GII**	**F^P^**
**SI**	14.20 ± 0.83^a^	11.80 ± 1.64^b^	5.202^0.0129^
**PL**	0.284 ± 0.015^a^	0.231 ± 0.027^b^	3.231^0.0039^
**AT**	4251.83 ± 16.87^a^	3542.51 ± 13.72^b^	4.402^0.0309^

* Means followed by the same letter did not differ significantly from each other by the Wilcoxon-Mann-Whitney test (P <0.05);

GI- group I (control); GII-group II (treated with metronidazole); SI- implantation site (7 days); PL- placenta (14 days); AT- total area; F^p^ – analysis of variance with probability of significance. SEM (standard error of the mean).

The implantation sites in the group of rats treated with placebo has been completely inserted into the wall of the uterus. Histologically, these sites were composed of trophoblasts, some with mitotic activity, polyploid cytotrophoblasts and rich vascularization. The luminal epithelium was characteristic of the simple columnar type and several endometrial glands were visualized in the decidua (Figure 1111D). The sites of implantation in the treated group presented the same histological characteristics than the control (Figure 2222D).

**Figure 1:**
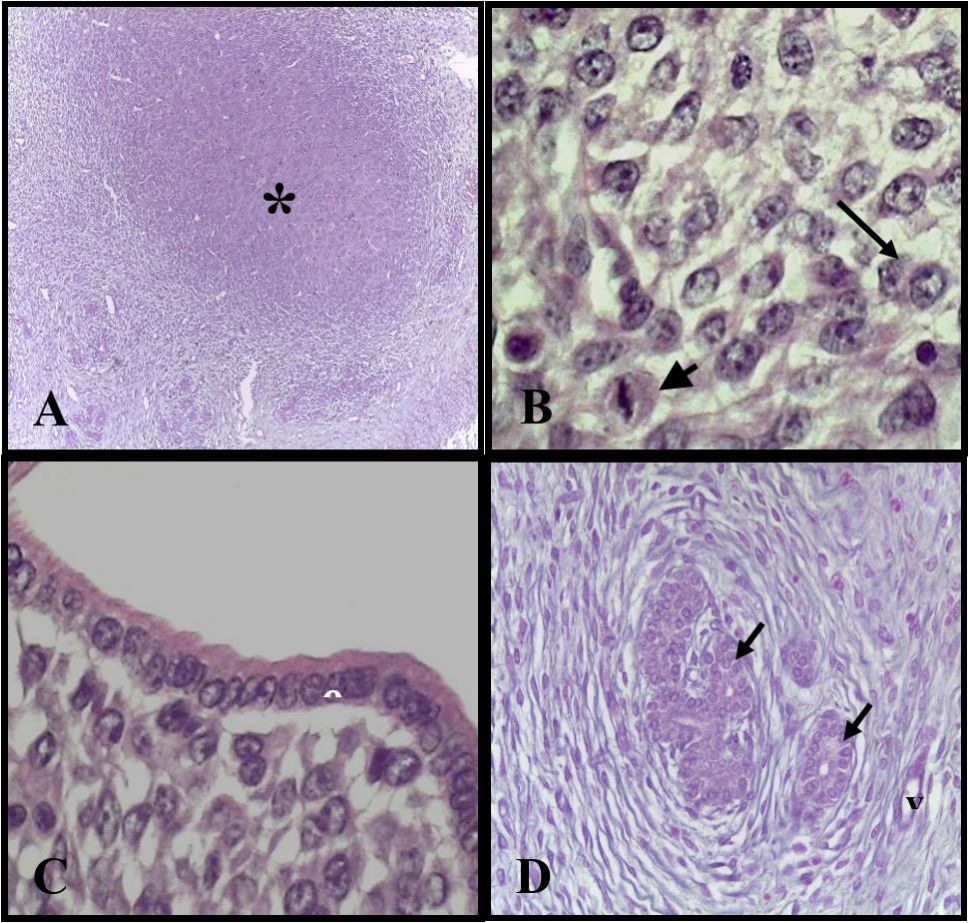
Photomicrography of rat uterus of the placebo group. (A) Implantation site (*) rats of the control group completely inserted in the uterus wall. H. E. ± 42X; (B) Trophoblasts in mitosis (minor arrow) and polyploid cytotrophoblast (major arrow). H.E ± 428X; (C) Luminal epithelium (el). ± 428X; (D) Blood vessels (vs) and endometrial glands (arrows). H.E ± 428X.

**Figure 2:**
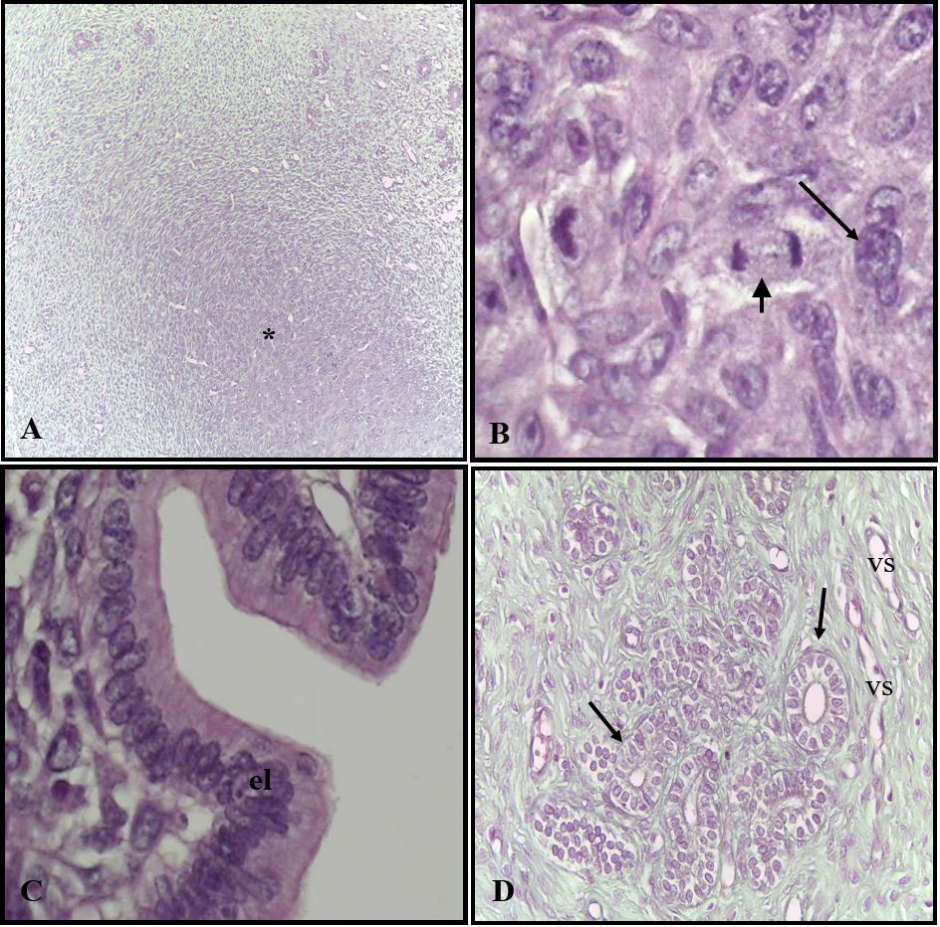
Photomicrography of the uterus of rats of the treated group. (A) Site of implantation (*) of rats of the treated group totally inserted in the wall of the uterus. H.E. ± 42X; (B) Trophoblasts in mitosis (minor arrow) and polyploid cytotrophoblast (major arrow). H.E ± 428X; (C) Luminal epithelium (el). H.E ± 428X; (D) Blood vessels (vs) and endometrial glands (short arrows). H.E. ± 428X.

There was a significant statistical reduction in placental weight in metronidazole-treated rats when compared to those in the placebo group (Table 1).

In the morphological analysis of the placentas at 14 days of development, both the animals from group I and II presented the same histological characteristics. The decidua region was highly vascularized and the placental disc’s region well developed, with the three layers: a) labyrinth layer, the outermost and thicker layer characterized by the presence of intervillous maternal blood spaces and fetal vessels; b) the spongiotrophoblast layer in which undifferentiated trophoblasts are observed; and c) the latter layer, which is formed by the giant trophoblastic cells; this layer blends with the decidua (Figure 333333F).

**Figure 3:**
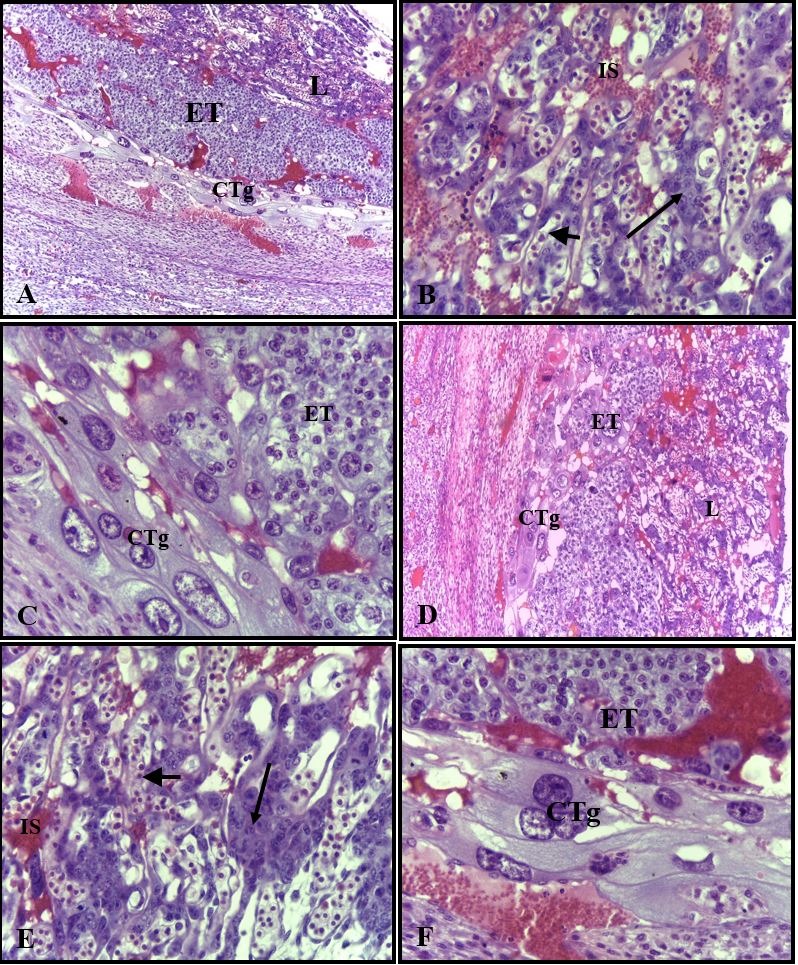
Photomicrography of placental disc of rats of the control group: (A) labyrinth (L), spongiotrophoblast (Tg) and giant trophoblastic cell (CTg). H.E. ± 107X; (B) Detail of labyrinth containing syncytiotrophoblast (major arrow), intervillous maternal blood spaces (Is) and fetal vessels (minor arrow). H.E. ± 428X; (C) Detail of spongiotrophoblast (Tg) and giant trophoblastic cells (CTg). H.E. ± 428X. Rat placental disc of the treated group: (D) labyrinth (L), spongiotrophoblast (Tg) and giant trophiblastic cell (CTg). H.E. ± 107X; (E) Detail of labyrinth containing syncytiotrophoblast (major arrow), intervillous maternal blood spaces (Is) and fetal vessels (minor arrow). H.E. ± 428X; (F) Detail of spongiotrophoblast (Tg) and giant trophoblastic cells (CTg). H.E. ± 428X.

Histochemical analysis revealed no significant changes in the content of collagen, elastic and reticular fibers (Table 2).

**Table 2:** Implantation site histochemistry (7 days) and placental disc (14 days) in the experimental groups*. Intense reaction (++), moderate (±) and weak (+).

	GI	GII	
Collagen fibers	++	++		
Elastic fibers	+	+		
Reticular fibers	±	±		

* GI- group I (control); GII-group II (treated with metronidazole).

Statistical analysis of total placental disc area means showed that group II presented a significant lowering compared to the control group (Table 1). In this group, significant differences were also observed in the labyrinth region for the syncytial trophoblasts, and maternal and fetal vascularization (Table 3). In the spongiotrophoblast layer, the mean of the trophoblastic cells and the syncytiotrophoblast were also significantly reduced compared to the control (Table 3).

**Table 3:** Constituent elements of the labyrinth region, spongiotrophoblast and giant trophoblastic cells of the placental disc with 14 days of development in the experimental groups. The results represent Means ±SEM of groups of 15 rats*.

	GI	GII	F^P^
IS	18.35 ± 1.16^a^	15.03 ± 1.18^b^	2.953^0.0285^
VF	15.26 ± 1.44^a^	12.32 ± 1.04^b^	3.388^0.0101^
TS	59.32 ± 2.09^a^	52.50 ± 2.33^b^	5.232^0.0041^
CT	42.18 ± 1.78^a^	39.15 ± 0.96^b^	2.315^0.0161^
ST	7.28 ± 0.15^a^	6.19 ± 0.67^b^	7.432^0.0402^
CTG	67.89 ± 2.34^a^	62.08 ± 2.24^a^	7.012^0.0619^

* Means followed by the same letter did not differ significantly from each other by the Wilcoxon-Mann-Whitney test (P <0.05);

GI- group I (control); GII-group II (treated with metronidazole); IS - intervillous maternal blood spaces; VF - fetal vessels; TS - syncytiotrophoblast; CT - trophoblast cells; ST – syncytiotrophoblast; CTG - giant trophoblast cells; F^p^- analysis of variance with probability of significance. SEM (standard error of the mean).

**Figure 4:**
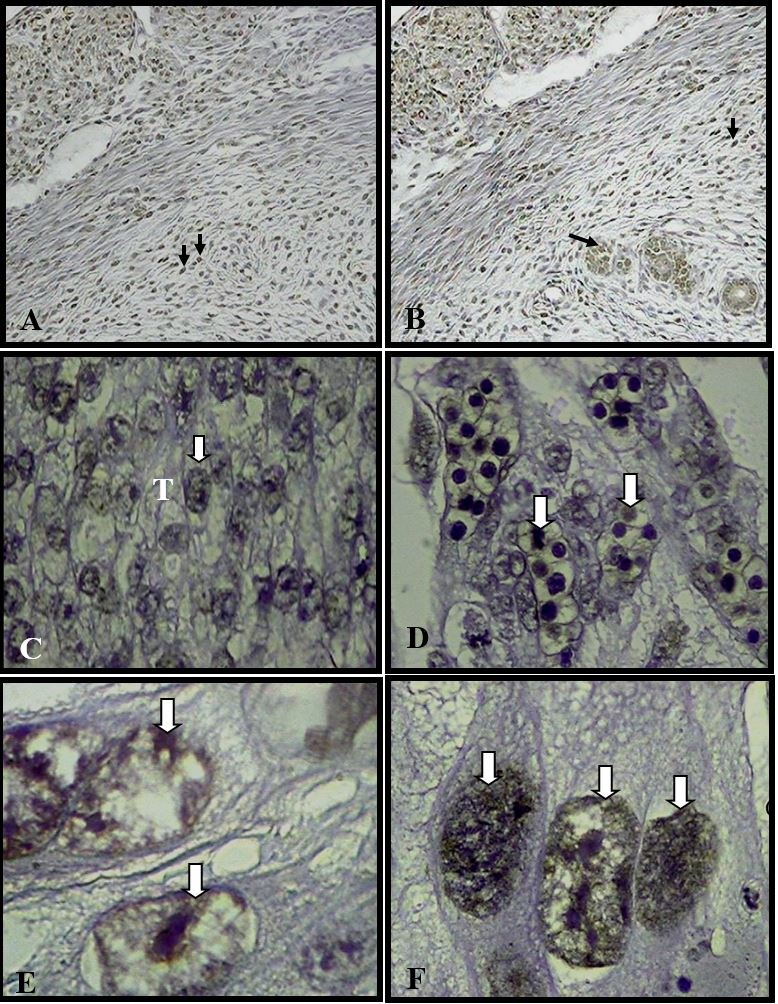
TUNEL Assay. Site of implantation and regions of the placental disc. (A) Site of the placebo group. ± 107X; (B) Site of implantation of the treated group. ± 107X; (C) Spongiotrophoblast (T) of the control group. ± 107X; (D) Labyrinth of the treated group. ± 107X; (E) Giant trophoblastic cells of the placebo group. ± 428X; (F) Giant trophoblastic cells of the treated group. ± 428X. Observe positive marking (arrow).

The TUNEL test showed apoptotic activity in the implantation sites and placentas at 14 days of development independent of the treatment (Figure 4).

There was no evidence of malformation in the head, trunk, and limbs of the neonates. However, there was a significant reduction in their number and weight in the group treated with metronidazole when compared to placebo without any effect on their length (Table 4).

**Table 4:** Weight (g) and length (cm) of the one-day-old pups born from the experimental groups. The results represent Means ±SEM of groups of 15 rats*.

	GI	GII	F^P^
Number	12.60 ± 0.54^a^	10.80 ± 1.30^b^	2.751^0.0325^
Weight	6.78 ± 0.^17a^	5.54 ± 0.58^b^	4.657^0.0105^
Length	6.22 ± 0.^31a^	6.12 ± 0.23^a^	1.702^0.0796^

* Means followed by the same letter did not differ significantly from each other by the Wilcoxon-Mann-Whitney test (P <0.05);

GI- group I (control); GII-group II (treated with metronidazole); F^p^- analysis of variance with probability of significance. SEM (standard error of the mean).

## Discussion

Despite having a broad antimicrobial spectrum against relevant vaginal pathogens (Mendling et al., 2019), metronidazole is a drug for which there are not enough data to characterize the potential risks for the embryo and/or fetus, thus limiting the opportunity of safe treatment of acute and chronic disorders during the gestational period.

When testing the hypothesis that metronidazole may present a noxious effect in the conception, a significant reduction in the number of implantation sites was observed in the rats treated with 130 mg/kg of metronidazole during the first seven days of gestation compared to group control. Embryo implantation is the process by which the embryo (blastocyst) acquires a stable position in the endometrium to enable the maintenance of an efficient system of metabolic exchanges between the endometrium and the embryo (Melo et al., 2014). In rat, this process occurs from the 1st to the 5th day of pregnancy (Medeiros et al., 2014), a period in which the blastocyst appears to be very sensitive to chemical exposures, which may even lead to its death (Bernardi and Spinosa, 2017). It is also known that in the pre-implantation phase, the embryo seems to be more susceptible to lethality than teratogenicity (Rodrigues et al., 2011). This suggests an effect of metronidazole on embryo pre-implantation action, since there was also a reduction in the number of neonates, but without reabsorption (death after implantation) and teratogenicity.

In addition, Mudry et al. (2001) also reported a decrease in the viability of embryos implanted in rats treated with metronidazole. These authors attributed this effect to the action of metronidazole on the ovarian follicles of the treated females, dismissing a post-fertilization effect since the administration of the drug occurred before mating. Furthermore, studies showed cytotoxicity of this drug in studies about germ cells (El-Nahas and El-Ashmawy, 2004; Rasheed et al, 2018), also indicating the possibility of a cytotoxic effect in some released oocytes.

Significant reduction in placental weight, total placental disc area, syncytiotrophoblast parameters, maternal and fetal vascularization in the labyrinth region, and the parameters of trophoblastic and syncytiotrophoblastic cells in the spongio-prophylactic region at 14 days of gestation in the treated group may be associated with the reduction in the gonadotrophic hormones, LH and FSH, levels. Because studies have shown that the intraperitoneal administration of metronidazole at a dosage of 200 and 400 mg/kg for 30 and 60 days led to a reduction in testosterone, FSH and LH levels in rats (Grover et al., 2001; Sohrabi et al., 2007).

Thus, alterations in the levels of these gonadotrophins, which play a synergistic role in the development of ovarian follicles, ovulation and implantation (Achache and Revel, 2006; Uribe-Velásquez et al., 2010), could lead to a decrease in the release of androgen, estrogen and progesterone. These hormones are responsible for the morphological and functional differentiation of the trophoblast during gestation, in addition to acting gradually on the basal layer of the endometrium, increasing its thickness due to mitosis and growth of the endometrial glands, stromal proliferation, vascular neoformation and spiral artery formation, which are important events for normal development of the placenta and, consequently, of the fetus (Zybina et al., 2001; Natale et al., 2003; Sutherland, 2003), which justifies the reduction in the weight of the offspring.

The apoptotic activity was observed at the implantation sites and placentas with 14 days of development independently of the treatment, therefore showing that treatment with metronidazole at a dose of 130mg/kg orally did not interfere with the natural apoptotic process of the placenta during gestation. Apoptosis occurs in the placenta throughout gestation, but the frequency increases in the final gestation period (Boss et al., 2003; Meça et al., 2010). This mechanism plays an important role in placental remodeling process, assisting in proper maintenance of tissue proportions (Smith et al., 1997; Correia-da-Silva et al., 2004) due to the fact that balance between proliferation and cell death is necessary for the success of the embryo implantation. This is important because the increased rate of apoptosis in decidual and trophoblastic cells may compromise placental function and, consequently, pregnancy success (Meça et al., 2010).

Regarding the histochemical analysis, there were no significant changes in collagen, elastic and reticular fibers contents in the sites and placentas of the treated group when compared to the control group. Several studies have demonstrated the action of metronidazole administered orally at a dose of 180mg/kg in the tissue repair process, accelerating wound contraction, collagen synthesis and epithelialization and promoting precocity in cicatrization by directly stimulating fibroblasts, the main cells involved in this process. However, higher doses of this drug may produce cytotoxicity in these cells (Rao et al., 2002; Girish and Patil, 2005), by preventing the synthesis of collagen, elastin, fibronectin, glycosaminoglycans and proteases necessary for tissue remodeling during healing (Amadeu et al., 2003; Hildebrand et al., 2005). Also, there are indications that metronidazole does not act directly on the fibroblasts, but rather diminishes the duration of the inflammatory phase, allowing the cells to speed up the healing process (Sampaio et al., 2009), which was reinforced by our study, since there were no sign of inflammatory processes in the implantation sites as well as in the placentas.

## Conclusion

The results observed in this study showed that 130 mg/kg metronidazole was harmful to gestation in rats, since it reduced the number of implanted embryos, the weight and the components of the placenta, in addition to the number and weight of neonates. This suggests that this drug should be used with caution during pregnancy, and it is necessary to ascertain whether the harmful effects of continuous exposure to this antibiotic could be extended to humans.
